# Real-world treatment persistence and predictive factors for discontinuation of antifibrotic therapies in patients with idiopathic pulmonary fibrosis: a *post-hoc* analysis of two multicenter observational cohort studies in Poland

**DOI:** 10.3389/fphar.2025.1586197

**Published:** 2025-06-05

**Authors:** Sebastian Majewski, Katarzyna Górska, Katarzyna B. Lewandowska, Magdalena M. Martusewicz-Boros, Małgorzata Sobiecka, Wojciech J. Piotrowski

**Affiliations:** ^1^ Department of Pneumology, Medical University of Lodz, Lodz, Poland; ^2^ Department of Internal Medicine, Pulmonary Diseases and Allergy, Medical University of Warsaw, Warsaw, Poland; ^3^ First Department of Lung Diseases, National Tuberculosis and Lung Diseases Research Institute, Warsaw, Poland; ^4^ Third Department of Lung Diseases and Oncology, National Tuberculosis and Lung Diseases Research Institute, Warsaw, Poland

**Keywords:** idiopathic pulmonary fibrosis, IPF, antifibrotic therapy, pirfenidone, nintedanib, treatment persistence, predictive factors, treatment discontinuation

## Abstract

**Background:**

Persistence with antifibrotic medications in patients with idiopathic pulmonary fibrosis (IPF) is crucial for long-term outcomes. However, real-world data regarding treatment persistence patterns in IPF are scarce.

**Methods:**

We conducted a *post hoc* analysis of two retrospective, real-world, multicenter observational studies (PolExPIR and PolExNIB) that collected clinical data on Polish patients with IPF managed at specialized centers between January 2017 and October 2021. We compared clinical variables between groups of patients who continued and discontinued antifibrotics and evaluated predictive factors for treatment discontinuation.

**Results:**

Overall, 808 patients were included in the analysis. Of these, 278 subjects (34.4%) discontinued therapy over a median follow-up of 16 (8–24) months. The proportion of patients discontinuing therapy was comparable between pirfenidone and nintedanib (37.5% vs. 32.5% respectively; p = 0.15). Additionally, no statistical difference was observed between antifibrotic agents in the distribution of time until treatment discontinuation (log-rank test, p = 0.3). Predictive factors associated with the probability of treatment discontinuation included age (hazard ratio [HR] 1.04; 95% confidence interval [CI] 1.02–1.05), body mass index (BMI, HR 0.97; 95% CI 0.94–0.99), transfer factor of the lung for carbon monoxide (TLco)% predicted (HR 0.98, 95% CI 0.97–0.99), Gender-Age-Physiology (GAP) index score (HR 1.3, 95% CI 1.18–1.42), use of long-term oxygen therapy (LTOT) (HR = 1.7, 95% CI 1.28–2.27) and intermittent dosing adjustment (HR 1.66, 95% CI 1.29–2.15).

**Conclusion:**

In this large population-based cohort of patients with IPF, around one-third discontinued antifibrotics during a study follow-up with no difference in the rates and time to discontinuation between pirfenidone and nintedanib. Clinical predictive factors including age, BMI, TLco% predicted, GAP index score, use of LTOT and intermittent dosing adjustment were associated with the risk of treatment discontinuation.

## 1 Introduction

Idiopathic pulmonary fibrosis (IPF) is a chronic, progressive, and irreversible lung disease with an inevitably fatal prognosis. Without treatment, the median survival time ranges from 3 to 5 years after diagnosis (Raghu et al., 2018). IPF cannot be cured, although two drugs with antifibrotic potency, pirfenidone and nintedanib, have been licensed for the treatment. Both agents are recommended as a standard of care for IPF pharmacotherapy by international and local guidelines (Raghu et al., 2018; Piotrowski et al., 2020). Pivotal randomized clinical trials (RCTs) have proven that antifibrotics can modify the natural history of IPF by reducing a decline of lung function (Noble et al., 2011; King et al., 2014; Richeldi et al., 2014) and showing survival benefit (Fisher et al., 2017; Lancaster et al., 2019; Noble et al., 2016; Richeldi et al., 2016). Therefore, persistence with antifibrotic medications in IPF is crucial and critical to slow disease progression and improve survival outcomes. Nevertheless, both RCTs and available real-world data studies report considerably high rates of discontinuations of antifibrotic agents, often related to poor tolerance, adverse drug reactions, or disease progression (King et al., 2014; Richeldi et al., 2014; Chaudhuri et al., 2014; Wijsenbeek et al., 2015; Harari et al., 2015; Hughes et al., 2016; Toellner et al., 2017; Galli et al., 2017; Antoniou et al., 2020; Harari et al., 2018; Dobashi et al., 2021). Moreover, data on predictive factors associated with the risk of discontinuing antifibrotics in IPF is limited. A better understanding of treatment persistence patterns and predictive factors associated with the risk of discontinuing antifibrotic agents could be valuable for clinicians providing care to patients with IPF.

The present study aimed to evaluate the rates of discontinuation of antifibrotic therapies and identify clinical predictive factors associated with discontinuation of treatment in a large population-based cohort of patients with IPF in Poland. This study utilized clinical datasets of patients from two recently published multicenter studies on Polish experiences with antifibrotic medications in IPF (PolExPIR and PolExNIB) (Majewski et al., 2020; Majewski et al., 2023).

## 2 Materials and methods

### 2.1 Study population and data collection

We performed a *post hoc* analysis of clinical datasets of Polish patients with IPF receiving antifibrotics in the frame of treatment program reimbursed by the National Health Fund (NHF) from two previously conducted multicenter observational real-world data studies (Majewski et al., 2020; Majewski et al., 2023). The PolExPIR study retrieved data on patients with IPF treated with pirfenidone (data collection from January 2017 to September 2019), whereas the PolExNIB study retrieved data on patients with IPF treated with nintedanib (data collection from March 2018 to October 2021). Briefly, the main inclusion criteria for the NHF treatment program were a multidisciplinary team diagnosis of IPF according to guidelines (Raghu et al., 2018; Piotrowski et al., 2020), forced vital capacity (FVC) ≥ 50% of predicted value, and transfer factor of the lung for carbon monoxide (TLco) ≥ 30% predicted value. The main exclusion criteria were compatible with contraindications to pirfenidone or nintedanib according to product characteristics. Moreover, subjects with FVC < 50% predicted or TLco < 30% predicted were not eligible for enrolment. Both observational studies assessed baseline and follow-up clinical data of patients initiating antifibrotic treatment at the participating centers to analyze real-world safety and efficacy. It is worth noting that in the PolExPIR study, none of the enrolled patients had been treated with nintedanib prior to initiating pirfenidone, as nintedanib became available in Poland nearly 18 months after pirfenidone. In contrast, in the PolExNIB study, 17% of enrolled subjects (n = 88) had previously received pirfenidone.

In the present study, we evaluated drug persistence data and clinical predictive factors associated with discontinuation of antifibrotics over the study follow-up. Both source studies, PolExPIR and PolExNIB, collected data on treatment discontinuations due to adverse drug reactions, disease progression, neoplastic disease, lung transplantation, death and other reasons. In the present study, we have focused on all-cause discontinuations within the combined patient cohort. The drug exposure period was defined as the time between the date of treatment initiation and the date of treatment discontinuation for any reason or end of study follow-up. Approval from the local ethics committee was not required as this study analyzed deidentified datasets obtained from two previously published studies (Majewski et al., 2020; Majewski et al., 2023).

### 2.2 Statistical analysis

All statistical analyses were performed with the use of Stata software, version 18.0 (StataCorp, College Station, Texas, United States). A mean with standard deviation (SD) was used to express continuous normally distributed data, while the median with interquartile range (IQR) was used for not normally distributed data. Absolute numbers and relative frequencies were used to express categorical data. Comparisons between independent groups were performed using an unpaired t-test (for normally distributed data) or Wilcoxon rank-sum test (for not normally distributed data) for continuous variables and a Chi-square test for binary variables. Kaplan-Meier analysis was used to evaluate the time to discontinuation of treatment and probabilities of discontinuation of antifibrotic therapy among patients receiving pirfenidone or nintedanib over the study follow-up. The hazard rates for treatment discontinuation between patients treated with pirfenidone and nintedanib were compared using the log-rank test. Additionally, patients starting treatment with pirfenidone and nintedanib were analyzed over the medication exposure period using a Cox proportional hazards regression models adjusted for confounding factors. The following clinical variables were considered as confounders: age, sex, body mass index (BMI), FVC% predicted, TLco% predicted, Gender-Age-Physiology (GAP) index score, use of long-term oxygen therapy (LTOT), time from symptoms to diagnosis, time from diagnosis to treatment and intermittent dosing adjustments. The results of Cox regression analyses are expressed as hazard ratios (HR) with 95% confidence intervals (95% CI) and p-values. Significance was accepted at a two-sided p-value < 0.05.

## 3 Results

### 3.1 Study population

A combined cohort of 808 patients with IPF was analyzed. The median follow-up time did not differ between patients receiving pirfenidone (17 [12–23] months) and nintedanib (15 [7–25.5] months, p = 0.68). Additionally, both subgroups of patients had similar baseline characteristics. However, a statistical analysis revealed that at the initiation of antifibrotic therapy patients starting treatment with pirfenidone were slightly younger, had slightly worse lung function as measured by FVC, were more often males, and had longer times for treatment initiation than patients starting nintedanib treatment (see Table 1).

**TABLE 1:** Baseline demographics and clinical characteristics of patients with IPF receiving antifibrotics in PolExPIR and PolExNIB cohorts.

Characteristic	Pirfenidone (n = 307)	Nintedanib (n = 501)	p-value
Age, mean (SD), years	68.8 (8.1)	70.1 (8)	0.002
Male, n (%)	237 (77.2)	349 (69.7)	0.02
BMI, mean (SD), kg/m^2^	28.4 (3.9)	28.2 (3.9)	0.35
FVC % predicted, median (IQR)	77.1 (67–88.5)	80.2 (68.5–95.1)	0.007
TLco% predicted, median (IQR)	52.2 (42.6–64.6)	55.9 (42.2–69.0)	0.08
GAP index, median (IQR), score	3 (3–4)	3 (3–4)	0.32
LTOT, n (%)	51 (16.6)	74 (14.8)	0.48
Symptoms to diagnosis time, median (IQR), months	15.5 (9.5–30)	12 (6–28)	0.11
Diagnosis to treatment time, median (IQR), months	6 (2–24)	3 (1–11)	0.001
Follow-up time, median (IQR), months	17 (12–23)	15 (7–25.5)	0.68
Intermittent dosing adjustment, n (%)	74 (24.1)	101 (20.2)	0.22

Abbreviations: BMI, body mass index; FVC, forced vital capacity; GAP index, gender, age, and two physiology variables (FVC and TL_CO_); LTOT, long-term oxygen therapy; TL_CO_, transfer factor of the lung for carbon monoxide.

### 3.2 Drug persistence

Over the median follow-up time of 16 (8–24) months for the combined cohort of patients, 278 subjects (34.4%) discontinued antifibrotic treatment. No statistical difference was noted in the rates of discontinuation between subjects receiving pirfenidone and nintedanib (37.5% vs. 32.5% respectively, p = 0.15). Similarly, the median time to discontinuation was not significantly different between antifibrotic agents (p = 0.06) (see Table 2).

**TABLE 2:** Persistence with antifibrotic medications in patients with IPF in PolExPIR and PolExNIB studies.

Characteristic	Pirfenidone (n = 307)	Nintedanib (n = 501)	p-value
Discontinuation, n (%)	115 (37.5)	163 (32.5)	0.15
Time to discontinuation, median (IQR), months	7 (4–13)	10 (4–16.7)	0.06

**FIGURE 1:**
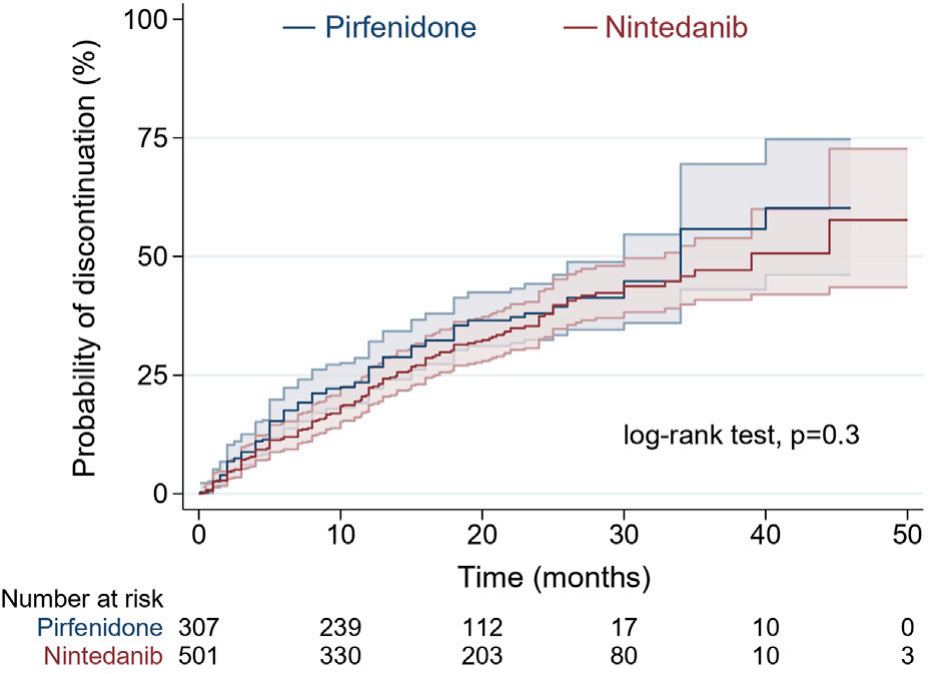
Probability of treatment discontinuation in patients starting pirfenidone (blue) and nintedanib (red) over medication exposure period by Kaplan-Meier analysis (shadow lines are reflecting 95% confidence interval).

No significant difference was noted between pirfenidone and nintedanib in terms of the distribution of time until discontinuation of treatment by the log-rank test (p = 0.3). A comparison of the probability of treatment discontinuation in patients starting pirfenidone and nintedanib over the medication exposure period is shown in Figure 1.

**FIGURE 2:**
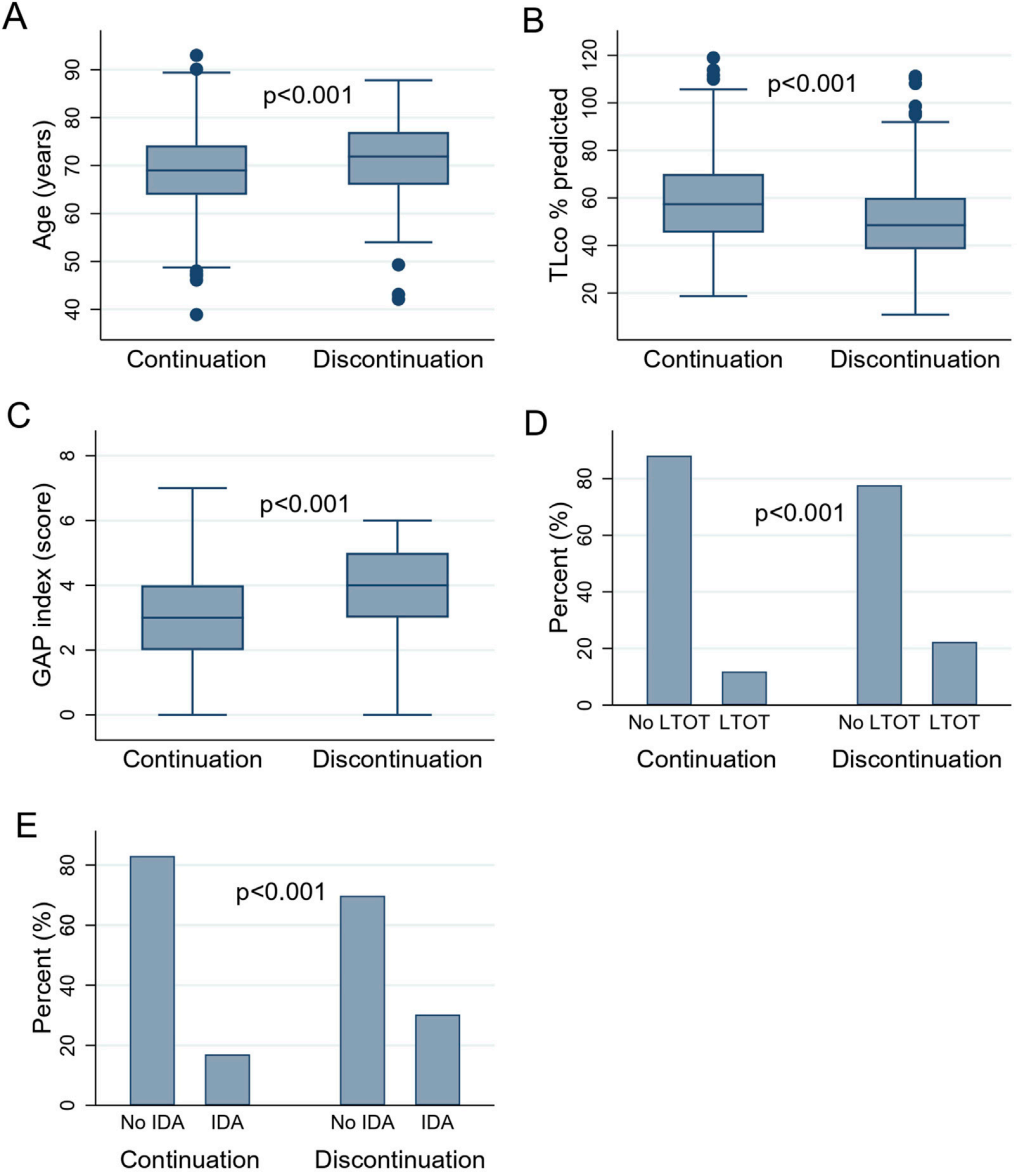
**(A–E)** presenting the difference in clinical variables in patients with IPF according to drug persistence status: **(A)** The difference in age of patients with IPF according to drug persistence status. **(B)** The difference in TLco% predicted of patients with IPF according to drug persistence status. **(C)** The difference in GAP index score of patients with IPF according to drug persistence status. **(D)** The difference in the use of LTOT in patients with IPF according to drug persistence status. **(E)** The difference in the IDA in patients with IPF according to drug persistence status. Abbreviations: IPF, idiopathic pulmonary fibrosis; TL_CO_, transfer factor of the lung for carbon monoxide; GAP index, gender, age, and two physiology variables (FVC and TL_CO_); LTOT, long-term oxygen therapy; IDA, intermittent dosing adjustment. Boxes represent medians and interquartile ranges, while whiskers represent the 5th and 95th percentiles.

Drug persistence analysis in relation to baseline characteristics showed that patients discontinuing treatment were older, had worse lung function expressed by lower TLco% predicted, had more advanced disease according to a higher GAP index score, and used LTOT more often than patients continuing treatment (p < 0.001 for all comparisons). Moreover, follow-up data revealed that patients discontinuing treatment needed more often intermittent dosing adjustments including temporary drug interruption and/or dose reduction (n = 84 out of 278; 30.2%) than patients continuing antifibrotics (n = 90 out of 530, 16.9%; p < 0.001), see Table 3 and Figure 2. The unadjusted hazard ratio (HR) for the probability of discontinuation of treatment in nintedanib-treated vs. pirfenidone-treated patients, using the Cox proportional hazard regression model was 0.88 (95% confidence interval (CI) 0.69–1.12, p = 0.3). After adjustment for confounding factors including age, sex, BMI, FVC%, TLco%, GAP index score, use of LTOT, time from symptoms to diagnosis, time from diagnosis to treatment, and intermittent dosing adjustment the HR for treatment discontinuation in nintedanib-treated versus pirfenidone-treated patients was 0.92 (95% CI 0.7–1.2, p = 0.53), see Table 4.

**TABLE 3:** Baseline demographics and clinical characteristics of patients with IPF according to treatment persistence status in a combined cohort of patients from PolExPIR and PolExNIB studies (n = 808).

Characteristic	Discontinuation (n = 278)	Continuation (n = 530)	p-value
Age, mean (SD), years	71.1 (8.2)	68.9 (7.5)	<0.001
Male, n (%)	203 (73)	383 (72)	0.82
BMI, mean (SD), kg/m^2^	28 (3.8)	28.4 (3.9)	0.15
FVC % predicted, median (IQR)	76.6 (67–91.9)	80.5 (68.4–92.4)	0.20
TLco % predicted, median (IQR)	48.6 (38.5–60.1)	57.4 (45.4–70.1)	<0.001
GAP index, median (IQR), score	4 (3–5)	3 (2–4)	<0.001
LTOT, n (%)	62 (22.3)	63 (11.9)	<0.001
Time from symptoms to diagnosis, median (IQR), months	12 (7–30)	13 (7–28)	0.95
Time from diagnosis to treatment, median (IQR), months	4.5 (1.5–15)	4 (1–13)	0.12
Time of treatment, median (IQR), months	8.9 (4–15)	20 (13–26)	<0.001
Intermittent dosing adjustment, n (%)	84 (30.2)	90 (16.9)	<0.001

Abbreviations: BMI, body mass index; FVC, forced vital capacity; GAP index, gender, age, and two physiology variables (FVC and TL_CO_); IPF, idiopathic pulmonary fibrosis; LTOT, long-term oxygen therapy; TL_CO_, transfer factor of the lung for carbon monoxide.

**TABLE 4:** Predictive factors for the discontinuation of antifibrotic therapy in a combined cohort of patients from the PolExPIR and PolExNIB studies.

Characteristic	Univariable analysis			Multivariable analysis		
	HR	95% CI	p-value	HR	95% CI	p-value
Pirfenidone treatment	1.0	—	—	1.0	—	—
Nintedanib treatment	0.88	0.69–1.12	0.3	0.92	0.7–1.2	0.53
Age	1.04	1.02–1.05	<0.001	1.04	1.02–1.06	<0.001
Sex (male)	0.99	0.76–1.3	0.97	0.91	0.65–1.27	0.59
BMI	0.97	0.94–0.99	0.03	0.98	0.95–1.01	0.25
GAP index	1.3	1.18–1.42	<0.001	1.09	0.92–1.29	0.30
FVC % predicted	0.99	0.99–1.0	0.35	0.99	0.99–1.00	0.85
TLco % predicted	0.98	0.97–0.99	<0.001	0.98	0.97–0.99	0.002
LTOT	1.7	1.28–2.27	<0.001	1.25	0.90–1.74	0.18
Time from symptoms to diagnosis	1.0	0.99–1.0	0.3	1.0	0.99–1.00	0.63
Time from diagnosis to treatment	1.0	0.99–1.0	0.94	0.99	0.98–1.00	0.22
Intermittent dosing adjustment	1.66	1.29–2.15	<0.001	1.49	1.12–1.98	0.005

Abbreviations: BMI, body mass index; GAP index, gender, age, and two physiology variables (FVC and TL_CO_); FVC, forced vital capacity; TL_CO_, transfer factor of the lung for carbon monoxide; LTOT, long-term oxygen therapy; HR, hazard ratio; CI, confidence interval; HRs, 95% CIs, and p-values were derived from unadjusted and adjusted Cox proportional hazard regression models were appropriate.

### 3.3 Predictive factors for discontinuation of antifibrotic therapy

**FIGURE 3:**
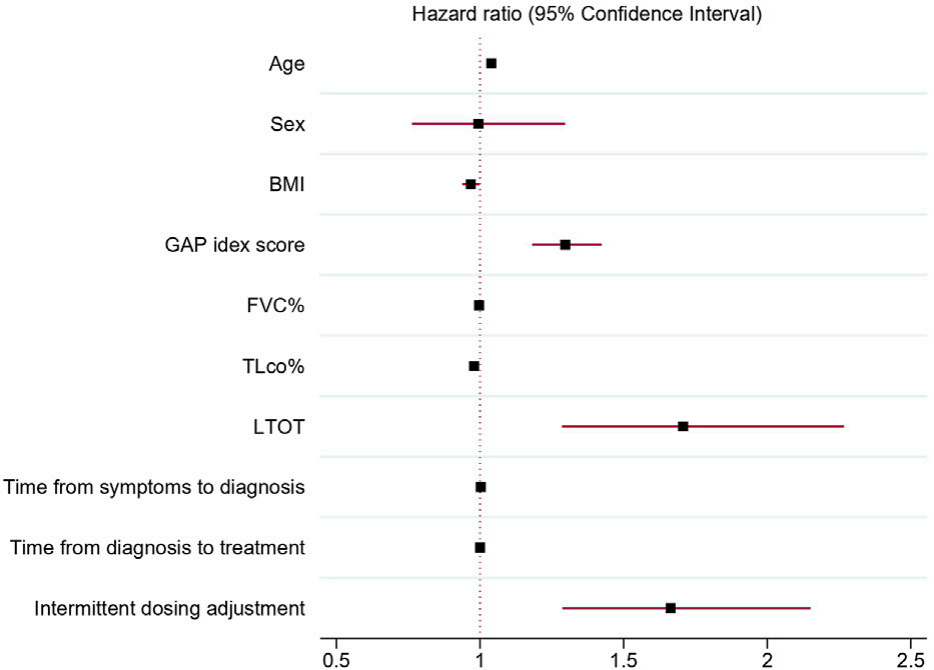
Forest plot showing hazard ratios and 95% confidence intervals obtained by univariable Cox proportional hazards regression models of independent clinical variables associated with antifibrotic treatment discontinuation. Abbreviations: BMI, body mass index; GAP index, gender, age, and two physiology variables (FVC and TL_CO_); FVC%, forced vital capacity % predicted; TL_CO_%, transfer factor of the lung for carbon monoxide % predicted; LTOT, long-term oxygen therapy.

**FIGURE 4:**
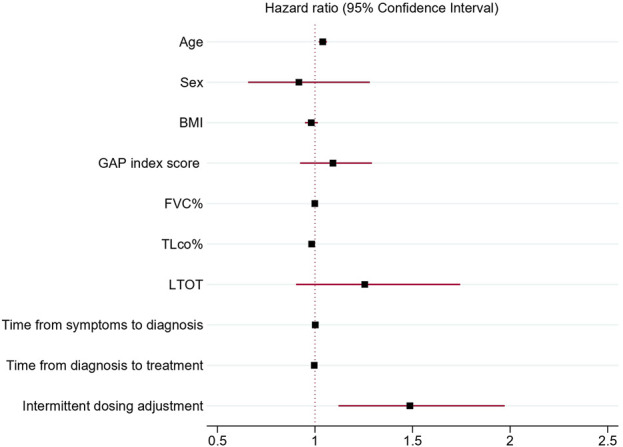
Forest plot showing hazard ratios and 95% confidence intervals obtained by multivariable Cox proportional hazards regression model of independent clinical variables associated with antifibrotic treatment discontinuation. Abbreviations: BMI, body mass index; GAP index, gender, age, and two physiology variables (FVC and TL_CO_); FVC%, forced vital capacity % predicted; TL_CO_%, transfer factor of the lung for carbon monoxide % predicted; LTOT, long-term oxygen therapy.

Association of discontinuation of antifibrotic treatment with independent clinical variables analyzed in univariable Cox proportional hazards regression models confirmed a significant association of age (HR 1.04; 95% CI 1.02–1.05 p < 0.001), BMI (HR 0.97; 95% CI 0.94–0.99, p = 0.03), TLco% predicted (0.98, 95% CI 0.97–0.99p < 0.001), GAP index score (HR 1.3, 95% CI 1.18–1.42, p < 0.001), use of LTOT (HR = 1.7, 95% CI 1.28–2.27,p < 0.001) and intermittent dosing adjustment (HR 1.66, 95% CI 1.29–2.15, p < 0.001) with discontinuation of antifibrotics. Moreover, the multivariable Cox proportional hazards regression model adjusted for confounding factors confirmed that age (HR = 1.04, 95% CI; 1.02–1.06, p < 0.001), TLco% predicted (HR = 0.98: 95% CI; 0.97–0.99, p = 0.002) and intermittent dosing adjustment (HR 1.49, 95% CI 1.12–1.98, p = 0.005) were independent clinical variables associated with the probability of discontinuation of treatment, see Table 4 and Figures 3, 4 for detailed results.

## 4 Discussion

The present study is the first nonregistry real-world analysis of antifibrotic medication persistence and clinical predictive factors associated with treatment discontinuation in patients with IPF in Poland. Our analysis showed that about one-third (34.4%) of patients discontinued antifibrotics over a median follow-up time of 16 months. Additionally, no significant difference in rates and time to discontinuation of therapy between pirfenidone and nintedanib was noted. We confirmed a significant association between age, BMI, TLco% predicted, GAP index score, use of LTOT and intermittent dosing adjustment with the risk of discontinuation of antifibrotic medications in univariable Cox regression models. Moreover, the multivariable Cox proportional hazards regression model identified age, TL_CO_%, and intermittent dosing adjustment as independent predictors of antifibrotic treatment discontinuation. Considering the importance of adherence to antifibrotic treatment for the prognosis of IPF, our findings might be valuable for clinicians providing care to patients with IPF.

The pathobiology of IPF involves repeated micro-injuries to alveolar epithelium caused by exposure to different noxious triggers. In genetically predisposed individuals, this leads to aberrant healing of alveolar epithelium, resulting in diffuse fibrosis of the lungs in the final stage of the disease (Lederer and Martinez, 2018; Baumgartner et al., 2000; Bédard Méthot et al., 2019; Trethewey and Walters, 2018; Majewski and Piotrowski, 2020). As a result of the pathogenic process, patients with IPF develop progressive dyspnea, cough, limitation of daily activities, impairment of quality of life, subsequent respiratory failure, and premature death. The inevitably fatal prognosis of IPF is characterized by a median survival of 3–5 years (Lederer and Martinez, 2018; Raghu et al., 2018). Despite years of efforts, no effective therapy for IPF was available and many studies aiming to develop safe and effective treatment have become failures over the last decades (Somogyi et al., 2019). Recently, two antifibrotic agents, pirfenidone and nintedanib, proved consistent around 50% reduction of lung function decline in a heterogeneous population of patients with IPF compared to placebo (Noble et al., 2011; King et al., 2014; Richeldi et al., 2014). None of the pivotal studies for antifibrotics was powered enough to show survival benefits, but pooled data analyses of RCTs, and several real-world data studies confirm that patients treated with antifibrotics live longer (Fisher et al., 2017; Lancaster et al., 2019; Noble et al., 2016; Richeldi et al., 2016; Kang et al., 2020).

To date, pirfenidone and nintedanib are the only treatment options recommended as standard of IPF pharmacotherapy (Raghu et al., 2018; Piotrowski et al., 2020). Although, there is no consensus which antifibrotic agent should be regarded as a first-line option. When making a therapeutic decision, the drug’s safety profile, any existing health conditions and concurrent treatments, lifestyle factors, and the patient’s preferences should be considered (Górska et al., 2022). Nevertheless, both agents slow the decline in lung function, therefore persistence with antifibrotic therapies is in the best interest of patients in terms of the long-term benefits. It is important to note, that in clinical practice, many patients with IPF discontinue treatment due to many reasons including but not limited to poor tolerance, adverse drug reactions, or disease progression (King et al., 2014; Richeldi et al., 2014; Chaudhuri et al., 2014; Wijsenbeek et al., 2015; Harari et al., 2015; Hughes et al., 2016; Toellner et al., 2017; Galli et al., 2017; Antoniou et al., 2020; Harari et al., 2018; Dobashi et al., 2021). In a recent study of the large IPF cohort (>1,300 patients) from France, nintedanib and pirfenidone treatment was discontinued within 12 months of initiation in 43.8% and 51.5% of patients, respectively (Belhassen et al., 2021). This population-based cohort study using digital data from the French National Health System confirms that the continuity of antifibrotic therapy is challenging in real-world clinical practice and many patients discontinue treatment losing the chance for the long-term benefits it can provide (Belhassen et al., 2021).

Recently, a few retrospective observational studies from Medicare beneficiaries and privately insured patients with IPF in the United States of America (United States) who initiated antifibrotics were published. In the first study (>3,500 patients with IPF), a mean follow-up in days was 204 and 189 for pirfenidone and nintedanib, and discontinuation rates were 24.6% and 29.1%, respectively (Corral et al., 2019). Interestingly, contrary to the findings of the French study, observed drug persistence was significantly higher in patients receiving pirfenidone than nintedanib. Although, the Medicare population in this study had a shorter observation time than the French cohort. Further analysis of the Medicare data would be necessary to determine whether such differences continue over a longer period. In the second study (2,901 treated patients), the discontinuation rate was 42.8% (not reported separately for each antifibrotic agent) over the mean treatment duration of 302 days (Dempsey et al., 2021). The most recent study (3,179 treated patients) reported the highest discontinuation rate of 72.1% (no separate data for each antifibrotic agent) within the first year since treatment initiation (Ramos et al., 2024). There are also several small and single-center observational studies with short follow-up duration reporting data on persistence with antifibrotic agents in heterogenous IPF cohorts available in the literature. However, the value of their findings has many limitations, and therefore are not discussed.

Noteworthy, our study had a longer follow-up than the above-mentioned studies reporting data on antifibrotic treatment persistence patterns. An interesting finding from our analysis is no significant difference in the rates and time to discontinuation between pirfenidone and nintedanib. Furthermore, despite longer follow-up period, discontinuation rates for either pirfenidone or nintedanib (37.5% and 32.5%, respectively) or any antifibrotic medication in the combined cohort (34.4%) were significantly lower than observed in the French or United States cohorts. The reasons for this discrepancy are not fully clear. Our cohort was smaller (808 patients) than the cohorts from France or the United States, and data collection differed (multicenter study collecting individual patients’ data from medical records at participating centers vs. medical database analyses). Nevertheless, these differences alone are unlikely to account for discrepancies in discontinuation rates.

One possible explanation for noted differences could be a greater motivation of Polish patients to continue treatment despite experiencing tolerance issues and adverse drug reactions. It is also likely, that treating physicians’ knowledge and experience in behavioral modifications, dose reductions, and temporary discontinuations were very effective strategies in allowing antifibrotic therapies to continue in a greater number of Polish patients. Moreover, the lower discontinuation rates observed in the Polish cohort could be explained by patients being under more specialist care in one of the ILD reference centers through which they have access to fully reimbursed antifibrotic medications in the setting of the treatment programs. It should be emphasized that reimbursed antifibrotic therapies available for patients with IPF in Poland require a prescription and treatment supervision by a pulmonologist. Such locally organized care of patients facilitates more careful monitoring and management in case of any issues with antifibrotic treatment. Lastly, full reimbursement of antifibrotics with no additional out-of-pocket (OOP) costs for patients with IPF meeting the inclusion criteria for enrolment to treatment programs in Poland could also account for noted differences in the discontinuation rates. This situation is particularly relevant in the case of patients from the United States, where OOP costs could partly explain the low adoption of antifibrotics (only one in four patients with IPF treated with antifibrotics) and could significantly influence antifibrotic treatment persistence (Dempsey et al., 2021). It is important to note that the proposed explanations are speculative, and hypothesis-generating, and cannot be confirmed based on the construction of retrospective observational studies and their limitations in this context. However, if at least part of the proposed explanations justifies lower discontinuation rates of antifibrotic medications observed in patients with IPF in Poland, this suggests well-organized access to antifibrotic therapies through a countrywide network of specialized ILD centers with experienced healthcare providers supervising IPF patients’ management and supporting patients in persisting with treatment.

The French real-world data study identified that older age, female sex, and use of supplemental oxygen were associated with a greater risk of discontinuation of antifibrotic treatment (Belhassen et al., 2021). Other studies found that clinical variables associated with the increased risk of discontinuation of antifibrotic therapies include older age (Galli et al., 2017; Wright et al., 2021; Bhomra et al., 2018), female sex (Wright et al., 2021; Bhomra et al., 2018; Romero Ortiz et al., 2024), BMI (Wright et al., 2021; Takehara et al., 2024), FVC < 60% predicted (Ogawa et al., 2018) and TLco < 40% predicted (Bhomra et al., 2018). Our pooled data analysis did not confirm that the female sex or FVC% were associated with the risk of discontinuation of antifibrotic therapies. However, our findings regarding the relationship between older age, BMI, TLco% predicted and use of LTOT with the risk of discontinuation of antifibrotics corroborate well with the previous findings and confirm the validity of our analysis. Furthermore, for the first time to our knowledge, we demonstrated that a higher GAP index score and intermittent dosing adjustment are significantly associated with a greater risk of discontinuation of antifibrotic medications. The multidimensional GAP index and staging system was originally developed to provide a simple screening method to determine the average risk of mortality of patients with IPF (Ley et al., 2012). It incorporates assessments of gender, age, and two physiology variables (FVC and TL_CO_) to calculate the total score and assign a GAP stage to predict the risk of mortality in IPF. According to our analysis, a one-point increase in the GAP index score increases the risk of discontinuation of antifibrotics by 30%. This data confirms that the GAP index can serve as a good predictor of discontinuation of antifibrotics in patients with IPF. Intermittent dosing adjustment, including temporary drug interruptions and/or dose reductions, are common therapeutic interventions for managing adverse drug reactions. These adjustments can enable the continuation of antifibrotic therapy. Our study results confirmed that patients needing intermittent adjustments are at greater risk for discontinuation of treatment. Noteworthy, in our study, the multivariable Cox proportional hazards regression model identified age, TL_CO_% and intermittent dosing adjustment as independent clinical variables significantly associated with the probability of antifibrotic treatment discontinuation. Identifying predictive factors for treatment discontinuation, along with predictors of treatment response, could improve patient management by minimizing unnecessary exposure to therapy that may be ineffective or cause significant side effects. However, biomarkers that are able to predict or assess treatment response in IPF can have an impact as part of precision medicine only if they reach clinical practice (Karampitsakos et al., 2024).

In summary, our findings may help clinicians identify patients with IPF who are at higher risk of treatment discontinuation and may benefit from closer monitoring, earlier intervention, and tailored support strategies. The results underscore the importance of proactive management, particularly in older patients and those with more advanced disease, to improve treatment persistence. Given the inevitable and poor prognosis of IPF, the lack of alternative treatment options, and the high rates at which current treatments are discontinued, there is an urgent need for new, more effective, and better-tolerated therapies for IPF.

Among observational studies reporting data on persistence with antifibrotic treatment in patients with IPF, our study is unique. The strengths of this study include the analysis of a large population-based cohort of IPF patients with a confirmed diagnosis according to international guidelines, and a long follow-up period. In Poland, the prescription of reimbursed antifibrotic agents is strictly controlled, and access to therapy is restricted for pulmonologists. Moreover, the requirement of diagnosis confirmation in the setting of the multidisciplinary discussion for each individual patient makes us confident that our study population consisted of patients with a valid diagnosis of IPF. Confirmation of diagnosis for each patient is not possible in the case of many databases or registry-based observational studies. In addition, the broad patient population and a long follow-up may record a more complete picture of real-world persistence with antifibrotics than smaller and shorter observational studies.

Although there are several strengths and important findings from this study, there are also limitations to be recognized. The most important limitations are intrinsic to retrospective, multicenter, observational cohort studies. Several possible biases should be considered due to RWD documentation that is not as robust as that of RCTs and can lead to missing data and reporting bias. The difference in number of patients enrolled in the PolExPIR and PolExNIB studies could be considered as a limitation in relation to the assessment of discontinuation rates between pirfenidone and nintedanib. Furthermore, the lack of survival data for patients discontinuing treatment makes analysis of survival between subjects continuing and discontinuing treatment not possible in this cohort. Comorbidities and concomitant medication could also influence drug persistence. Source data studies were not collecting such information, therefore precise analysis of this aspect was not possible. Despite the above limitations, our findings extend current knowledge on the real-world persistence patterns with antifibrotics and provide novelty in our understanding of clinical predictive factors associated with the risk of discontinuing antifibrotic treatment in patients with IPF.

## 5 Conclusion

In a large real-world cohort of patients with IPF from a national network of specialized ILD centers, 34.4% discontinued antifibrotic therapies over a median follow-up of 16 months. There was no significant difference between pirfenidone and nintedanib in terms of discontinuation rates or time to discontinuation. Clinical predictive factors including age, BMI, TLco% predicted, GAP index score, use of LTOT and intermittent dosing adjustment were associated with the risk of discontinuation of antifibrotic medications in univariable Cox proportional hazard models. Notably, age, TL_CO_% predicted and intermittent dosing adjustment remained independently associated with treatment discontinuation in the multivariable analysis. Our study findings may assist clinicians caring for patients with IPF in identifying those who need closer treatment monitoring, earlier interventions, and strategies to support continued therapy. Moreover, our study extends the current understanding of treatment persistence patterns in IPF and suggests a well-organized and supervised IPF management system in Poland.

## PolExPIR and PolExNIB studies

The Authors thank the following co-investigators who have contributed to the PolExPIR and PolExNIB studies. Adam Barczyk and Marzena Trzaska-Sobczak (Department of Pneumonology, School of Medicine in Katowice, Medical University of Silesia, Katowice), Halina Batura-Gabryel and Agata Nowicka (Department of Pulmonology, Allergology and Pulmonary Oncology, Poznan University of Medical Sciences, Poznan), Adam J. Białas and Karolina Szewczyk (Department of Pneumology, Medical University of Lodz, Lodz), Małgorzata Buchczyk, Dariusz Jastrzębski and Dariusz Ziora (Department of Lung Diseases and Tuberculosis, School of Medicine with the Division of Dentistry in Zabrze, Medical University of Silesia, Katowice), Anna Doboszyńska and Luiza Grabowska-Skudlarz (Department of Pulmonology, University of Warmia and Mazury in Olsztyn, Pulmonology Hospital, Olsztyn), Hanna Jagielska-Len (Clinical Department of Lung Diseases, K. Marcinkowski University Hospital, Zielona Gora), Agnieszka Jarzemska (Department of Rapid Pulmonary Diagnostics, Kuyavian and Pomeranian Pulmonology Centre, Bydgoszcz), Ewa Jassem (Department of Pneumonology, Medical University of Gdansk, Gdansk), Marek Koprowski (Department of Civilization Diseases and Lung Diseases, John Paul II Specialist Hospital, Cracow), Michał Krawczyk (1st Department of Lung Diseases and Respiratory Allergy, Voivodeship Center for Lung Disease Treatment and Rehabilitation, Lodz), Rafał Krenke (Department of Internal Medicine, Pulmonary Diseases and Allergy, Medical University of Warsaw, Warsaw), Jan Kuś, Beata Żołnowska and Witold Tomkowski (1st Department of Lung Diseases, National Tuberculosis and Lung Diseases Research Institute, Warsaw), Barbara Mackiewicz and Janusz Milanowski (Department of Pneumonology, Oncology and Allergology, Medical University of Lublin, Lublin), Małgorzata Noceń-Piskorowska (Department of Tuberculosis and Pulmonology, West Pomeranian Voivodeship Hospital, Szczecin), Kazimierz Roszkowski-Śliż and Elżbieta Wiatr (3rd Lung Diseases and Oncology Department, National Tuberculosis and Lung Diseases Research Institute, Warsaw), Alicja Siemińska (Department of Allergology, Medical University of Gdansk, Gdansk), Aleksander Kania, Krzysztof Sładek and Tomasz Stachura (Department of Pulmonology, Jagiellonian University Medical College, Cracow), Małgorzata Tomczak (Department of Pulmonology, E.J. Zeyland Wielkopolska Center of Pulmonology and Thoracic Surgery, Poznan).

## Data Availability

The data that support the findings of this study are available from the corresponding author upon reasonable request. Requests to access these datasets should be directed to sebastian.majewski@umed.lodz.pl.
